# The natural product biosynthetic potential of Red Sea nudibranch microbiomes

**DOI:** 10.7717/peerj.10525

**Published:** 2021-02-04

**Authors:** Samar M. Abdelrahman, Nastassia V. Patin, Amro Hanora, Akram Aboseidah, Shimaa Desoky, Salha G. Desoky, Frank J. Stewart, Nicole B. Lopanik

**Affiliations:** 1 School of Earth and Atmospheric Sciences, Georgia Institute of Technology, Atlanta, GA, USA; 2 Faculty of Science, Suez University, Suez, Egypt; 3 School of Biological Sciences, Georgia Institute of Technology, Atlanta, GA, USA; 4 Center for Microbial Dynamics and Infection, Georgia Institute of Technology, Atlanta, GA, USA; 5 Faculty of Pharmacy, Suez Canal University, Ismailia, Egypt; 6 Department of Microbiology and Immunology, Montana State University, Bozeman, MT, USA

**Keywords:** Nudibranch, Microbiome, Natural products, Natural product biosynthesis

## Abstract

**Background:**

Antibiotic resistance is a growing problem that can be ameliorated by the discovery of novel drug candidates. Bacterial associates are often the source of pharmaceutically active natural products isolated from marine invertebrates, and thus, important targets for drug discovery. While the microbiomes of many marine organisms have been extensively studied, microbial communities from chemically-rich nudibranchs, marine invertebrates that often possess chemical defences, are relatively unknown.

**Methods:**

We applied both culture-dependent and independent approaches to better understand the biochemical potential of microbial communities associated with nudibranchs. Gram-positive microorganisms isolated from nudibranchs collected in the Red Sea were screened for antibacterial and antitumor activity. To assess their biochemical potential, the isolates were screened for the presence of natural product biosynthetic gene clusters, including polyketide synthase (PKS) and non-ribosomal peptide synthetase (NRPS) genes, using PCR. The microbiomes of the nudibranchs were investigated by high-throughput sequencing of 16S rRNA amplicons.

**Results:**

In screens against five model microorganisms, 51% of extracts displayed antimicrobial activity against more than one organism, and 19% exhibited antitumor activity against Ehrlich’s ascites carcinoma. Sixty-four percent of isolates contained PKS and NRPS genes, suggesting their genomes contain gene clusters for natural product biosynthesis. Thirty-five percent were positive for more than one class of biosynthetic gene. These strains were identified as belonging to the Firmicutes and Actinobacteria phyla via 16S rRNA gene sequencing. In addition, 16S rRNA community amplicon sequencing revealed all bacterial isolates were present in the uncultured host-associated microbiome, although they were a very small percentage of the total community. Taken together, these results indicate that bacteria associated with marine nudibranchs are potentially a rich source of bioactive compounds and natural product biosynthetic genes.

## Introduction

Increasing occurrences of antibiotic-resistant bacterial infections (Gootz, 2010), and high mortality rates from cancer, the second most lethal disease in the world (Siegel, Miller & Jemal, 2018), suggest that novel antibiotic and anticancer drugs are urgently needed. Marine ecosystems harbor high levels of biodiversity in animal and microbial communities, and are an important source of bioactive chemical compounds. Some of these molecules have been developed into pharmaceuticals, such as Ziconotide, an analgesic peptide discovered from a tropical cone snail, and the anticancer drug Yondelis from the sea squirt *Ecteinascidia turbinata* (McGivern, 2007). Further, spongothymidine and spongouridine isolated from the sponge *Tectitethya crypta* led to the development of the anti-leukemia drug cytarabine (Molinski et al., 2008; Lindequist, 2016; Altmann, 2017). Marine invertebrates, including nudibranchs, are thus important potential sources of drug discovery.

Compounds isolated from marine invertebrates include those with antimalarial, anti-inflammatory, antiviral and anticancer activity (Senthilkumar & Kim, 2013; Blunt et al., 2018). Nudibranchs (Mollusca, Gastropoda) lack a shell, are often brightly coloured and slow moving, and must protect themselves against various predators, such as fish and crabs, as well as against pathogenic microorganisms. Nudibranchs also produce mucus with high lipid and protein content to clean their surface and protect themselves from prey nematocysts; as these lipids and proteins represent a highly desirable food source for microbes, bioactive compounds may protect the host’s epidermis from pathogens (Wahidullah et al., 2006). Chemical ecology studies have shown many nudibranchs possess defensive secondary metabolites, often found in the dorsum, which is exposed to potential predation (Thompson et al., 1981; Mudianta et al., 2014; Dean & Prinsep, 2017; Blunt et al., 2018; Winters et al., 2018). These metabolites often are sequestered from food sources such as sponges, tunicates, cnidarians, and bryozoans. For instance, some nudibranchs accumulate alkaloids, diterpenes, and sesquiterpenes obtained from sponges (Bogdanov et al., 2016; Mudianta et al., 2016; Winters et al., 2018). These compounds can also be incorporated into egg masses, to help protect the eggs from predators (Pawlik et al., 1988). In some cases, however, nudibranchs appear to produce natural products on their own (Proksch, 1994; Dean & Prinsep, 2017). A study on the biosynthesis of secondary metabolites found in *Candlina luteomarginata* showed that certain terpenoids are synthesized de novo by the animal (Kubanek, Graziani & Andersen, 1997).

While microorganisms have been implicated in the production of natural products in many other marine invertebrates (Anjum et al., 2016), the possibility of a microbial source of nudibranch natural products has not been considered until relatively recently (Riyanti, Widada & Radjasa, 2009; Bohringer et al., 2017). Microbial associates have been shown to produce natural products previously attributed to their hosts for many invertebrate taxa, including sponges, cnidarians, ascidians, and bryozoans (Davidson et al., 2001; Lopanik, Lindquist & Targett, 2004; Piel et al., 2004; Schmidt et al., 2005). Despite the diversity of metabolites isolated from nudibranchs and microorganisms associated with marine invertebrates, few studies have documented the natural product potential of nudibranch microbiomes. For instance, extracts from *Pseudoalteromonas* and *Marinomonas* strains isolated from Indonesian nudibranchs possessed antimicrobial activity against methicillin-resistant *Staphylococcus aureus* COL (MRSA) and *Escherichia coli* O-19592 (EHEC) respectively. Another study on Indonesian nudibranchs found that two bacterial isolates had anti-MRSA activity (Kristiana et al., 2020). However, while cultivation has yielded microbial strains potentially capable of producing natural products, the larger potential of nudibranch communities as a source of novel compounds remains underexplored.

In this study, we examined the microbial natural product biosynthetic potential of four different nudibranch species collected in the Red Sea using culture-dependent and independent methods. We focused on Gram-positive bacteria as they are prolific producers of natural products (Genilloud, 2017). Bacteria were isolated from homogenates of these nudibranchs and screened for anticancer and antibiotic activity as well as natural product biosynthetic genes. Those isolates that possessed some measure of bioactivity and biosynthetic gene fragments were identified based on 16S rRNA gene sequence. Finally, the microbial communities of the nudibranchs were investigated using high-throughput sequencing to place the isolates in the ecological context of the microbial community. This comprehensive approach of coupling traditional cultivation with deep pyrosequencing of 16S rRNA gene amplicons allowed us to compare the bacterial communities of four different co-occurring nudibranch species, as well as determine the relative abundance of the cultivated bacterial isolates. Moreover, microbiomes of marine invertebrates remain poorly represented in the literature and in publicly available databases, and thus our data constitute a valuable addition to the study of host-microbiome associations.

## Materials and Methods

### Nudibranch collection and processing

**Figure 1:**
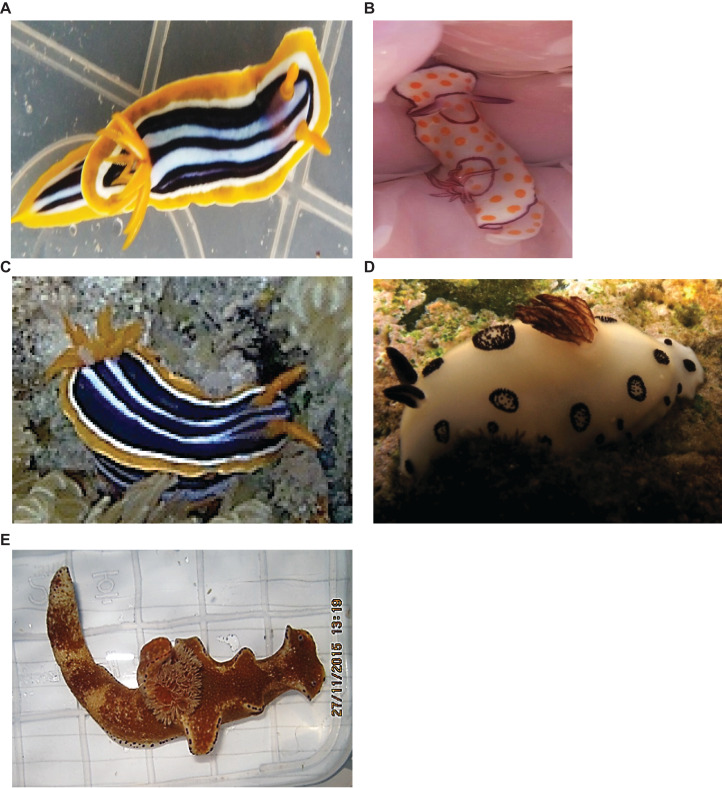
Nudibranch species used in this study. (A) *Chromodoris quadricolor*, (B) *Goniobranchus annulatus*, (C) *Chromodoris africana*, (D) *Jorunna funebris* (Image credit: Hugues Flodrops, http://www.seaslugforum.net/showall/jorufune) and (E) *Ceratosoma trilobatum*.

Nudibranchs were collected by SCUBA in the Red Sea near El Tor in the Gulf of Suez, Egypt (28.2278°N, 33.6211°E and 28.0856°N, 33.6822°E) at water depths between one and eight meters (Faculty of Pharmacy, Suez Canal University, Research Ethical Committee approval number 201511R1). Five animals belonging to different species (Fig. 1) were hand-picked while wearing latex gloves. As animals were collected in two expeditions (11/2015 and 05/2017), it was not possible to perform all experiments on all five species. Individuals were housed in sterilized containers with aerated seawater and maintained at ambient seawater temperature until they were transferred to the laboratory. Two samples (~4 gm) from each animal were directly homogenized for microbial isolation (see below) and two were preserved in DNA/RNA stabilization buffer for subsequent DNA extraction.

### Identification of nudibranchs

Preliminary visual identification of the collected nudibranchs was performed using the guide “Nudibranchs of the World” (Debelius & Kuiter, 2007). For molecular identification, genomic DNA was isolated from individual nudibranchs stored in DNA/RNA stabilization solution using the C-TAB method (Panova et al., 2016) or the *Quick*—DNA Fungal/Bacterial Miniprep Kit (Zymo Research, Irvine, CA, USA). Partial mitochondrial cytochrome oxidase *I* (*COI*) gene sequences were amplified using LCO1490-JJ and HCO2198-JJ primers (Astrin & Stüben, 2008) in a 20 μL PCR with Phire Green Hot Start II DNA Polymerase (Fisher Scientific, Waltham, MA, USA). Each PCR contained Phire Green Reaction Buffer, 10 mM dNTPs (New England Biolabs, Ipswich, MA, USA), 20 mg/ml BSA, 10 pmol of each primer and 1u Phire Hot Start II DNA Polymerase. The thermocycling conditions were as follows: initial denaturation was performed at 98 °C for 30 s followed by 35 cycles of denaturation 98 °C for 5 s, annealing at 55 °C for 5 s, extension at 72 °C for 7 s with a final extension at 72 °C for 1 min. Resulting amplicons were visualized by agarose gel electrophoresis, column-purified (DNA Clean & Concentrator-5 Kit; Zymo Research, Irvine, CA, USA), and sequenced using the PCR primers. Resulting sequences were queried against the NCBI nr database using BLAST to identify the nudibranch species. The partial mitochondrial *COI* gene sequences for the nudibranchs have been submitted to the NCBI under accession numbers MT446216, MT446217, MT639354 and MT659206.

### Isolation of associated bacteria

Gram-positive bacteria were first isolated from three nudibranchs species (*Ceratosoma trilobatum*, *Chormodoris quadricolor* and *Jorunna funebris*) collected in November 2015. After it was noted that a high proportion of the extracts from the isolates had bioactivity, additional nudibranch specimens were collected (*Goniobranchus annulatus*, *Chormodoris quadricolor* and *J. funebris*) in May 2017 to produce more isolates. Each nudibranch was rinsed three times with filter-sterilized natural sea water (NSW) to remove debris and unattached bacteria. Nudibranchs were cut into pieces with a sterile razor blade and then homogenized in a blender. The homogenate was serially diluted with NSW and spread (100 μL) on solid Marine Agar media, R2A, Actinomycetes Isolation Agar (AIA), starch casein agar, ISP2 agar and M1 agar. All media were prepared using NSW except Marine Agar, which was prepared with distilled water and 20% NaCl. All media were supplemented with 100 μg/mL cyclohexamide and 25 μg/mL nystatin to suppress fungal growth, and 25 μg/ml nalidixic acid to inhibit the growth of aggressive Gram-negative bacteria (Webster & Hill, 2001). Plates were incubated in the dark at 30 °C for two months until colonies appeared. Morphologically distinct isolates from the six different media from each animal were selected for purification and streak-plated on agar until the colonies appeared to be axenic.

### Extraction of secondary metabolites

Single colonies were inoculated into R2A liquid media and incubated for 3–5 days in a rotary shaker at 150 rpm at 30 °C to generate starter cultures. These were used to inoculate 50 mL R2A liquid media in Erlenmeyer flasks, which were then incubated at 30 °C while shaking at 150 rpm until the cultures reached stationary phase, usually 5 days. The cultures were transferred to a −20 °C freezer for 24 h. An equal volume of ethyl acetate was added to the liquid cultures, mixed (150 rpm, 1 h at room temperature), and transferred to a separatory funnel to separate the organic phase. This ethyl acetate extraction was performed three times. The organic phase solvent was evaporated and the residue was weighed and dissolved in DMSO to achieve a concentration of 5 mg/mL.

### Screening of antimicrobial activity

The first group of bacterial isolates (100) were screened for antimicrobial activity using the standard well diffusion assay (Flemer et al., 2012). Crude metabolic extracts (50 µL of 5 mg/mL) were tested against standard pathogens (OD at ƛ650 nm = 0.45) plated on Muller Hinton agar medium. Model microorganisms used to assess antimicrobial activity were the bacteria *S. aureus* ATCC 25923, *Escherichia coli* ATCC 25922, *Bacillus subtilis* ATCC 6633, and the fungus *Candida albicans* ATCC 10231. Equivalent volumes of DMSO and ethyl acetate were used as negative controls. Following incubation at 37 °C overnight, resultant zones of inhibition were measured.

### Screening for antitumor activity against Ehrlich ascites carcinoma cells

Ehrlich’s ascites carcinoma (EAC) cells collected from donor female mice (Swiss albino) (National Cancer Institute, Cairo University, Egypt) were suspended in sterile isotonic saline (0.9%). The viability of the cells was 99% as determined by the trypan blue assay, according to the method described in (McLimans et al., 1957). EAC cells (12.2 × 10^6^ cells/ml phosphate buffer) were treated with bacterial metabolic extracts (500 µg/mL) in DMSO. After 120 min of incubation at 37 °C, 50% trypan blue was added to an aliquot of the EAC mixture. The number of cells that showed signs of damage by stain penetration was counted using a hemocytometer with light microscope. The bacterial metabolic extracts that exhibited antitumor activity at 500 µg/mL were also tested at 50 and 250 µg/mL in DMSO using the same protocol.

### Isolate DNA extraction

Genomic DNA of the bacterial isolates (all 196) was extracted using the *Quick*—DNA Fungal/Bacterial Miniprep Kit (Zymo Research, Irvine, CA, USA). A single colony was inoculated into five mL of R2A broth and incubated in a shaker 150 rpm at 30 °C for 2–5 days depending on culture turbidity. Pelleted cells were resuspended in the recommended volume of Bashing Bead Buffer. Proteinase K was added and the cells incubated at 65°C for 1 h to increase cell lysis. Thereafter, the DNA extraction was carried out according to the manufacturer’s protocol for actinomycetes. Aliquots of the isolated DNA were visualized after gel electrophoresis and stored at −20 °C.

### Amplification of natural product biosynthetic genes

The bacterial isolates were screened for genes involved in the biosynthesis of secondary metabolites, specifically polyketide synthase (PKS; type I and type II) and non-ribosomal peptide synthetase (NRPS). Genomic DNA extracted from each isolate (20–50 ng/μL) was used as template for the amplification of the PKS-I, PKS-II and NRPS biosynthetic genes by PCR. Six sets of degenerate primers (Table 1) were used to amplify β-ketosynthase (KS) domain fragment within Type I and II PKSs. The presence of NRPSs was assessed by amplification of the conserved adenylation (A) domain using two sets of degenerate primers. All PCRs were performed using Phire Green Hot Start II DNA Polymerase using the same reaction concentrations as described above (nudibranch CO*I* amplification), but with DMSO instead of BSA. The thermocycling conditions were as follows: an initial denaturation at 98 °C for 30 s followed by 35 cycles of denaturation 98 °C for 5 s, annealing *T*_m_ and time depending on the primer sequence, extension at 72 °C for 10 s, followed by a final extension at 72 °C for 2 min. The amplicons were visualized following gel electrophoresis in 1% agarose.

**Table 1:** Existing and newly designed primers used in this study.

Target gene	Primer	Sequences	Approximate amplicon length (bp)	References
PKSI	K1F	TSAAGTCSAACATCGGBCA	1,200–1,400	Ayuso-Sacido & Genilloud (2005)
	M6R	CGCAGGTTSCSGTACCAGTA		
PKSI	KSDPQQF	MGNGARGCNNWNSMNATGGAYCCNCARCANMG	700	Ayuso-Sacido & Genilloud (2005)
	KSHGTGR	GGRTCNCCNARNSWNGTNCCNGTNCCRTG		
PKSI	MDPQQRF	RTRGAYCCNCAGCAICG	750	Kim & Fuerst (2006)
	HGTGTR	VGTNCCNGTGCCRTG		
PKSII	PF6	TSGCSTGCTTGGAYGCSATC	600	Metsa-Ketela et al. (1999)
	PR6	TGGAANCCGCCGAABCCGCT		
NRPS	MTF	CCNCGDATYTTNACYTG	750–1,000	Tambadou et al. (2014)
	MTR	GCNGGYGGYGCNTAYGTNCC		
NRPS	A3F	GCSTACSYSATSTACACSTCSGG	700	Ayuso-Sacido & Genilloud (2005)
	A7R	SASGTCVCCSGTSCGGTAS		
16S rRNA	27f	AGAGTTTGATCMTGGCTCAG	1,400–1,600	Chen et al. (2015)
	1492r	ACGGYTACCTTGTTACGACTT		
16S rRNA	331f	TTCTACGGGAGGCAGCAGT	460	Chan et al. (2018)
	797r	GGACTACCAGGGTATCTAATCCTGTT		
COI	LCO1490-JJ	GGTCAACAAATCATAAAGATATTGG	708	Astrin & Stüben (2008)
	HCO2198-JJ	TAAACTTCAGGGTGACCAAAAAATCA		
Unidentified bacteria	1F	TACATAGGGTGCGAGCG	220	This study
	220R	GTCAGTACATTCCCAGTTAGT		
	207R	CAGTTAGTTGCCTTCGCCATT		
Gamma-proteobacteria (A)	44F	GTGCGTGGCGGCATGAT	145	This study
	179R	TCCCGATATCTACGCATTC		
Gamma-proteobacteria (B)	41F	AGCGCACGTAGGTGGTGCGG	180	This study
	222R	TGTCAGTTACAGTCCAGGTGT		
	56F	TGCGGTAAGCCAGATGTGAAA	180	This study
	233R	TCGCTCCTCAGTGTCAGTTA		

### Bacterial 16S rRNA amplification and taxonomic assignment

Sixty-eight bacterial isolates (out of 196 total) tested positive for more than one type of biosynthetic gene (NRPS, PKS-I and PKS-II). These 68 isolates were identified based on their 16S rRNA gene sequence. Genomic DNA was used as a template to amplify approximately 1,400 base pairs of the 16S rRNA gene using the universal bacterial primers 27f and 1492r (Ehrenreich, Waterbury & Webb, 2005). All PCRs were performed using Phire Green Hot Start II DNA Polymerase, 10 mM dNTPs, 20 mg/ml BSA, 10 pmol of each primer and 1u Phire Hot Start II DNA Polymerase. The thermocycling conditions were as follows: initial denaturation was performed at 98 °C for 30 s followed by 35 cycles of denaturation 98 °C for 5 s, annealing at 56 °C for 5 s, extension at 72 °C for 12 s with a final extension at 72 °C for 1 min. The amplicons were purified using the DNA Clean & Concentrator Kit (Zymo Research, Irvine, CA, USA) and were used as template for bi-directional Sanger sequencing. Amplicons that yielded poor sequencing results were ligated into pGEM-T vector (Promega, Madison, WI, USA) and transformed into *Escherichia coli* DH5α. Plasmids were isolated from overnight cultures of colonies using the PureYield Miniprep kit (Promega, Madison, WI, USA). The insert was Sanger sequenced from both directions using the T7 forward and M13 reverse primers. The full 16S rRNA gene was assembled in Geneious Prime 2019.0.4 (USA). The taxonomy of the strains was determined using BLAST against the NCBI database based on the top BLAST hit. The partial 16S rRNA gene sequences for isolated bacteria have been submitted to the NCBI under accession numbers MT393617–MT393684.

### DNA extraction for microbiome analysis

Nudibranch specimens preserved in DNA/RNA stabilization buffer were transported to the laboratory and stored at −20 °C until processing. The nudibranchs were transferred to 4 °C overnight and washed with fresh RNA stabilization solution. Four individual nudibranchs were used for community analysis, representing the species *Chromodoris africana, Chormodoris quadricolor, G. annulatus* and *J. funebris*. In addition, *Chromodoris africana* was not collected in initial sampling expeditions, and therefore, there are no microbial isolations from it. A specimen collected later in September 2017, however, was used for the microbial community analysis as a comparison to its congener, *Chromodoris quadricolor*. The microbial community analysis was not performed on *Ceratosoma trilobatum* due to limited sample material. Each animal was cut longitudinally into two pieces and the skin and gut were separated for each half, resulting in four samples from each animal. The tissues were homogenized in liquid nitrogen, and genomic DNA was extracted according to the C-TAB protocol (Panova et al., 2016). Extracted DNA was visualized by agarose gel electrophoresis and quantified using a Nanodrop One spectrophotometer (Thermo Scientific, Waltham, MA, USA).

### Amplicon library preparation and sequencing

Illumina MiSeq libraries were prepared by amplifying the V4 region of the 16S rRNA gene using the environmental DNA protocol adopted from (Kozich et al., 2013). Briefly, amplicons were generated using Platinum^®^ PCR SuperMix (Life Technologies, Carlsbad, CA, USA) with Earth Microbiome Project primers 515F (Parada) and 806R (Apprill) appended with Illumina-specific adapters (Apprill et al., 2015; Parada, Needham & Fuhrman, 2016). All PCRs were performed using Phire Green Hot Start II DNA Polymerase (Fisher Scientific, Waltham, MA, USA) and BSA. Thermal cycling consisted of denaturation at 98 °C (30 s), followed by 30 cycles of denaturation at 98 °C (5 s), primer annealing at 55 °C (5 s) and primer extension at 72 °C (8 s), followed by extension at 72 °C for 1min. Amplicons were analyzed by gel electrophoresis to verify size (~400 bp, including barcodes and adaptor sequences) and purified using Diffinity RapidTip2 PCR purification tips (Diffinity Genomics, West Chester, PA, USA). Amplicons from different samples were pooled at equimolar concentrations and sequenced using a paired-end Illumina MiSeq 500 cycle kit with 5% PhiX to increase read diversity.

### Bioinformatic analyses and ecological statistics

Paired-end Illumina reads were trimmed with Trimmomatic-0.36 (Bolger, Lohse & Usadel, 2014) using the default leading and trailing parameters, a quality control sliding window of 4 bases with a minimum q-score cutoff of 25, and a minimum length cutoff of 150 bases. Forward paired reads were merged into one fasta file and run through QIIME2 (Bolyen et al., 2019) with the Deblur algorithm (Amir et al., 2017) to assess community composition. The use of Deblur meant sequences were not clustered into operational taxonomic units, but rather that each unique read was treated as a distinct taxon, hereafter referred to as a sequence variant (SV). Taxonomy was assigned using the SILVA 129 database (99% identity level). Quality filtered reads from all time points were submitted to the NCBI Sequence Read Archive (SRA) under BioProject PRJNA629797. Alpha diversity rarefaction values for each sample were generated in QIIME2 using Faith’s Phylogenetic Diversity metric using the “qiime diversity alpha rarefaction” command and a sequence depth cutoff of 2,318. SV read and taxonomic assignment tables were exported from QIIME2 and used with the Phyloseq (McMurdie & Holmes, 2015) and DivNet packages (Willis & Martin, 2018) in R to run beta diversity analyses at the SV level using the Bray–Curtis metric.

### Investigation of unidentified bacterial 16S rRNA gene sequences

Specific primers were designed for unidentified sequences from several individual SVs grouped under these identifiers that could not be classified beyond the domain level as “Bacteria” (Table 1) to confirm that these SVs were not sequencing artifacts. DNA from all four nudibranchs was amplified using these primers and Phire Green Hot Start II DNA Polymerase (Fisher Scientific, Waltham, MA, USA). The thermocycling conditions consisted of initial denaturation at 98 °C for 30 s followed by 35 cycles of denaturation 98 °C for 5 s, annealing *T*_m_ depending on the primer sequence for 5 s, extension at 72 °C for 2 s, and a final extension at 72 °C for 1 m. The amplified products (~170–200 bp in size) were visualized after gel electrophoresis in 2% agarose. PCR amplicons of the correct size were purified using the DNA Clean & Concentrator-5 kit (Zymo Research, Irvine, CA, USA) and sequenced in both directions. Sequences were aligned with nudibranch-associated microbiome sequences using Geneious followed by BLASTn searches against the NCBI nr database. In order to obtain longer sequences for more accurate identification, the specific primers were used in PCRs with universal bacterial 16S rRNA gene primers (331F and 797R) and nudibranch metagenomic DNA. Resulting PCR amplicons were sequenced in both directions. The taxonomy of the three individual SVs were determined using BLAST against the NCBI database based on the top BLAST hit.

## Results

### Nudibranch identification

Four nudibranchs out of five were identified using both morphological characteristics and DNA barcoding with *COI* gene sequences (Table 2). The fifth individual was identified based on morphology as *Ceratosoma trilobatum*, but limited material prevented *COI* analysis. The nudibranchs in this study represent five species from four genera: *Ceratosoma trilobatum, Chromodoris africana*, *Chromodoris quadricolor*, *G. annulatus* and *J. funebris*.

**Table 2:** Molecular identification of nudibranchs in this study.

Closest CO *I* gene BLAST match	Accession no.	Sequence position	Sequence size (bp)	Identity (%)
*Chromodoris africana* voucher CASIZ 194068	MG883098	1,552–2,046	494	98.0
*Chromodoris quadricolor* voucher CASIZ	MG883319	1,490–2,157	667	98.6
*Goniobranchus annulatus* voucher CHIhG1	KF408221	1,490–2,153	663	99.8
*Jorunna funebris* voucher CPIC 00633	KP871645	1,569–1,988	419	100.0

### Isolation of nudibranch-associated bacteria

A total of 196 bacterial isolates differentiated by colony morphology were obtained from the four nudibranch species in two rounds of isolation on six media types. The first isolation from *J. funebris*, *Ceratosoma trilobatum* and *Chormodoris quadricolor* homogenates produced 100 isolates. Once the high level of isolate extract bioactivity was observed (see below), another 96 isolates were obtained from homogenates of *J. funebris*, *Chormodoris quadricolor* and *G. annulatus*. *Chormodoris quadricolor* yielded the highest number of isolates. Most of the isolates were obtained on Marine Agar (32%), followed by R2A (27%), and M1 (17%). Nine percent were isolated on SCA and ISP2 and six percent from AIA.

### Screening for antimicrobial and antitumor activity

The metabolic extracts of the first 100 isolates (obtained in round 1) were tested for inhibitory activity against three model bacteria (*S. aureus* ATCC 25923, *Escherichia coli* ATCC 25922, *B. subtilis* ATCC 6633), and one fungus (*Candida albicans* ATCC 10231). Fifty-one isolates exhibited antimicrobial activity against at least one tested organism and 19 isolates displayed antimicrobial activity against two or more organisms (Table 3). Three isolates of *Streptomyces* spp. and one *Bacillus* sp. possessed antimicrobial activity against all tested pathogens. Seventeen of the 100 tested isolates exhibited antitumor activity against EAC cells; 16 of these also exhibited antibacterial activity. Extracts from two actinomycete isolates displayed the most antitumor activity against EAC with IC_50_ less than 300 µg/mL, while nine bacterial isolates (four Actinomycetes and five Firmicutes) had intermediate antitumor activity against EAC (IC_50_ less than 600 µg/mL).

**Table 3:** Percentage of isolate extracts with antimicrobial and antitumor activity.

Nudibranchl	Total no. of isolates	Antimicrobial bioactivity (%)	Antitumor bioactivity (%)	Isolates with antimicrobial and antitumor activity (%)
*Ceratosoma trilobatum*	24	54	12.5	12.5
*Chromodoris quadricolor*	55	51	13	9
*Joruna funebris*	21	47	33	33
Total	100	51	17	15

### Detection of PKS and NRPS biosynthetic genes fragments in isolates

Because a large proportion of the initial 100 isolates appeared to produce bioactive compounds, 96 more isolates were obtained, and all 196 were screened for the presence of natural product biosynthetic gene clusters. Sequences of the conserved KS and A domains of PKSs and NRPSs, respectively, were amplified from isolate genomic DNA by PCR using six sets of degenerate primers (Table 1). Sixty-four percent of the isolates showed positive PCR amplification for both KS and NRPS domains. The Type I KS domain successfully amplified in 48% of the isolates, and the Type II KS domain amplified in 39% of the isolates. Fifty percent of the isolates were positive for NRPS A domains. Of the strains with positive PCR amplification, 26% had only a single type of biosynthetic gene cluster, while the remaining positive strains had two or more types of biosynthetic genes: 2% had both PKS-I and PKS-II; 13% had both PKS-I and NRPS, 15% had both PKS-II and NRPS, and 43% of the positive strains had fragments of all three biosynthetic gene clusters (Table 4).

**Table 4:** Isolate identification, presence of natural product gene fragments (polyketide synthase types PKS I, PKS II and non-ribosomal peptide synthetase NRPS), and bioactivity of crude extracts in antimicrobial and antitumor assays.

Nudibranch	Strain	Closest 16SrRNA gene match	Accession no.	Identity (%)	PCR screening			Bioactivity*	
					PKS I	PKS II	NRPS	Anti- bacterial	Anti- tumor
*Joruna funebris*	1AS	*Nocardiopsis* sp. AE46	JF319150	99.91	+	–	+	++	+++
	2AS	*Nocardiopsis dassonvillei* subsp. albirubida VTT E-062983	EU430536	100.0	+	+	+	+++	++
	8AS	*Bacillus safensis* strain HA 527	KJ535336	99.8	–	+	+	+	–
	18AS	*Bacillus* sp. strain HBS1	MK966451	99.9	–	+	+	++	++
	24AS	*Bacillus flexus* strain CPS1.1	MT299668	99.85	–	+	–	+	+
	25AS	*Staphylococcus lentus* strain HS1-MRL	KX128918	100.0	+	+	–	+	+
	16A	*Bacillus aerius* strain FL101	KY819007	99.4	+	–	+		
*Ceratosoma trilobatum*	2BY	*Streptomyces xylophagus* strain TY190-20	MT083969	100.0	–	+	+	+	–
	4BY	*Bacillus* sp. S21722	KF956683	99.8	–	+	+	–	–
	6By	*Bacillus safensis* strain P9	MK210556	99.42	+	+	+	–	–
	11By	*Bacillus paramycoides* strain MMB	MT122838	99.88	–	+	+	+	–
	12BY	*Bacillus* sp. 6063	JX566648	99.9	+	+	+	++	–
	13BY	*Nocardiopsis dassonvillei* subsp. albirubida strain OAct926	MG661750	98.8	+	+	+	+++	+++
	15BY	*Bacillus* sp. S4713	JQ819877	99.9	–	+	+	++	+
	16BY	*Kocuria* sp. strain YKFH1121	MH298696	99.8	+	+	+	+	–
	23M1	*Nocardiopsis dassonvillei* strain XY236	MH432693	100	+	+	+	++	+++
*Chromodoris quadricolor*	6	*Bacillus safensis* strain SH10	MT256302	99.9	–	+	+	++	–
	9	*Bacillus* sp. strain C2-8	MT255139	100	+	+	+	+	–
	12	*Bacillus altitudinis* strain EB39	MT256105	100	+	–	+	+	+++
	14	*Oceanobacillus iheyensis* strain S6	MN056009	99.7	+	–	+	+	–
	16	*Bacillus australimaris* strain 96J27	MT192471	100	+	–	+	++	–
	17	*Bacillus safensis* strain NS3	KP279980	99.64	–	+	+	+++	++
	20	*Staphylococcus lentus* strain Hanna37	MN399938	99.64	+	–	+	+	–
	22	*Pseudomonas stutzeri* strain OsEnb_ALM_B7	MN889324	10	+	+	+	+	–
	24	*Planococcus ruber* strain CD8	MK216760	99.83	+	+	+	++	–
	31	*Bacillus amyloliquefaciens* strain VBS03	MG660863	99.85	–	+	+	–	+
	34	*Streptomyces* sp. E5N406	CP029624	99.7	+	+	+	++	+++
	38	*Staphylococcus lentus* strain PL445	MK015783	99.93	+	–	–	+	–
	41	*Enterococcus* sp. M190262	CP040461	99.93	+	+	+	–	–
	43	*Rhodococcus erythropolis* strain I-A-R-27	KT922050	99.3	+	+	+	+	–
	47	*Bacillus* sp. strain 22DM7	MK134623	99.8	+	+	+	–	–
	48	*Oceanobacillus kimchii* MXR1709B02	MN176502	99.8	+	+	–	–	–
	49	*Bacillus* sp. strain 1RM6 - 0	MK134620	99.8	+	–	+	–	–
	53	*Firmicutes bacterium* 00YDJ	EU180852	99.8	+	–	+	+++	–
	57	*Bacillus velezensis* strain 9D-6	CP020805	99.4	+	+	+	+++	++
	59	*Streptomyces qinglanensis* strain A5	MF682454	99.6	+	+	+	+++	–
	63	*Streptomyces* sp. SCSIO 001680	JQ031555	100	+	+	+	+++	+
	66	*Streptomyces* sp. B24AT	KU382279	100.0	+	+	+	+	–
	80	*Bacillus pumilus* strain B6	KJ870186	99.45	+	+	+	+	–
	2R	*Nocardiopsis* sp. I-Gauze-W-10-6	FJ267558	99.85	+	+	+		
	4R	*Streptomyces* sp. Ahbb4	KM214828	99.6	+	+	+		
	7R	Bacterium strain BH8S5	MT254881	99.93	–	+	+		
	8R	*Actinomycetales bacterium* XJSS-18	EU598254	100.0	+	+	+		
	2N	*Bacillus flexus* strain S72	MN005931	100.0	–	+	+		
	10N	*Staphylococcus lentus* strain HM17	MN401134	99.7	–	+	+		
	1AIA	*Oceanobacillus iheyensis* strain CN6-12	MH762168	99.6	+	+	+		
	4AIA	*Staphylococcus* sp. strain BH1-3	MN410649	100	+	+	+		
	3ISP2	*Bacillus siamensis* strain ICMP 20282	MF682396	99.7	+	+	+		
	6M1	*Bacillus safensis* strain SPa22LB	MT052639	100.0	+	+	+		
	10M1	*Bacillus* sp. strain 9RM71	MK134622	99.7	+	+	+		
	14M1	*Oceanobacillus iheyensis* HTE831	NR075027	99.8	+	+	+		
	15M1	*Bacillus* sp. HNA3	CP040881	100	+	+	+		
	16M1	*Streptomyces* sp. E2N173	KX279582	99.6	+	+	+		
	17M1	*Bacillus altitudinis* strain IHB B1644	MT328683	100.0	+	–	+		
	18M1	*Streptomyces* sp. strain MJM15241	MN636769	100.0	+	+	+		
	1S.C	*Staphylococcus* sp. SC1	KC951997	99.7	+	+	+		
	3S.C	*Nocardiopsis dassonvillei* strain NRK12	MH598413	99.8	–	+	+		
	7 S.C	*Staphylococcus lentus* strain PL444	MK015780	100.0	+	+	–		
*Goniobranchus annulatus*	15(2)	*Bacillus* sp. strain ZX1	MT305953	100.0	–	+	+		
	23(2)	*Kocuria* sp. strain C12-38	MT255185	100.0	+	+	+		
	25(2)	*Bacillus zhangzhouensis* strain 1.4517	MT316475	100.0	+	+	+		
	31(2)	*Halobacillus hunanensis* strain JSM 071077	LT714153	100.0	–	+	+		
	32(2)	*Bacillus safensis* strain T13	MT313116	100.0	+	–	+		
	34(2)	*Kocuria carniphila* strain NF28	MT262515	99.9	+	+	+		
	36(2)	*Kocuria carniphila* strain YM	JX485387	100.0	+	+	+		
	47(2)	*Bacillus* sp. strain YB-3	MF661927	100.0	–	+	+		
	59(2)	*Nocardiopsis* sp. 06-St-023	GU574127	99.9	+	+	+		
	78(2)	*Bacillus subtilis* strain RI4914	CP051306	100.0	–	+	+		

**Note:**

* For antimicrobial activity, zone of inhibition: + <10 mm; ++ ≤15 mm; and +++ ≤ 20. For antitumor activity, IC_50_: measured with µg/mL, + > 700; ++ < 700 µg/mL; +++ < 400 µg/mL. − = no activity.

### Molecular identification of nudibranch-associated bacteria

Sixty-eight isolates that tested positive for more than one of the classes of natural product biosynthetic gene fragments (PKS-I, PKS-II, NRPS) were identified by their 16S rRNA gene sequence (Table 4). The isolates included members overwhelmingly of the Firmicutes, primarily *Bacillus*, *Staphylococcus* and *Oceanobacillus* and Actinobacteria, mainly *Streptomyces*, *Nocardiopsis* and *Kocuria*. Identity ranges from 96.1% to 100% compared to top BLAST matches in GenBank. As expected, many isolates that were positive for natural product biosynthetic gene fragments, as well as antibacterial and antitumor activity, were Actinobacteria.

### Microbiome diversity and composition

**Figure 2:**
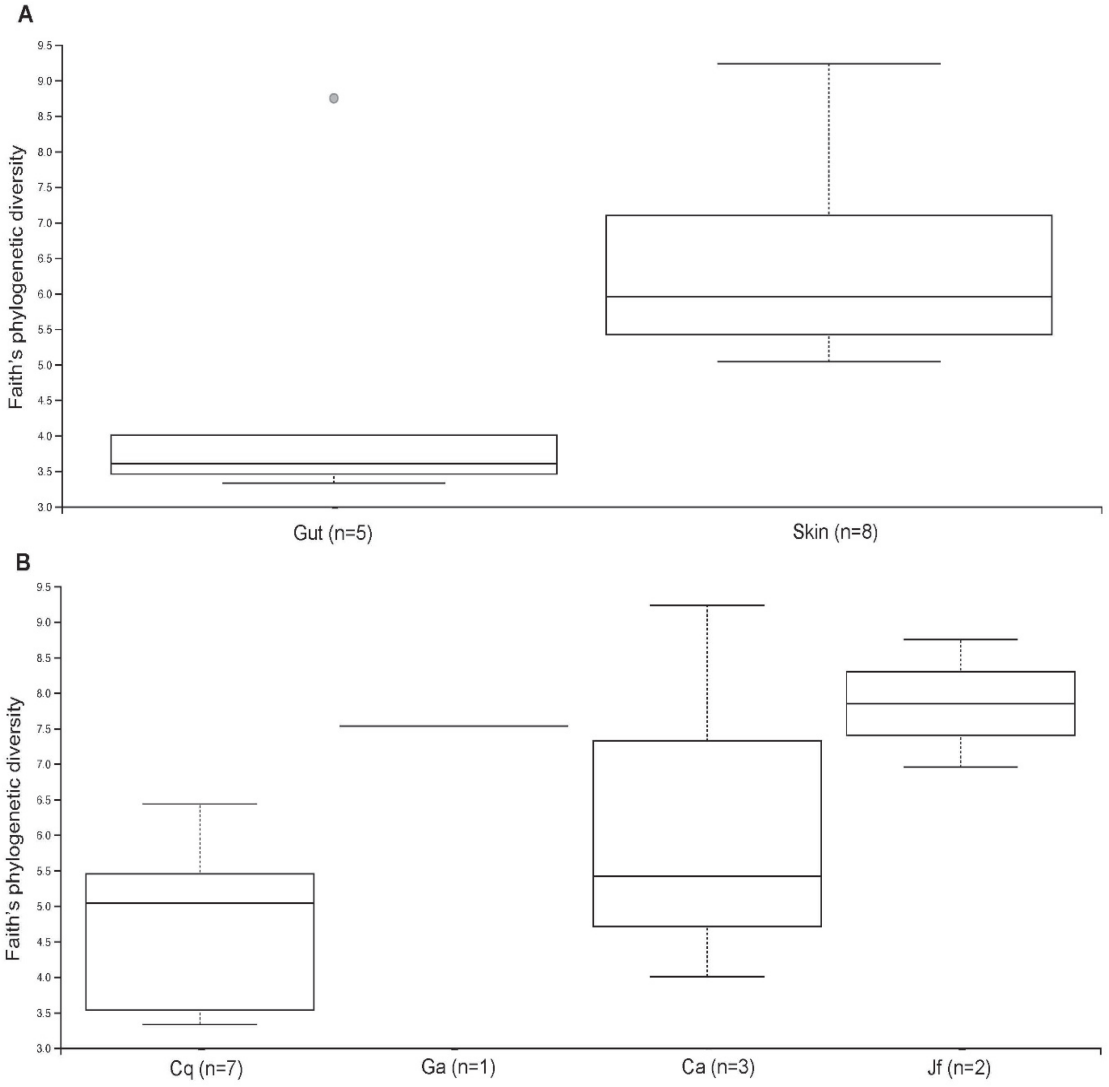
Alpha diversity of microbiomes in different nudibranch specimens and tissues. Alpha diversity differed by (A) body site (skin vs. gut) and (B) host organism (Cq = *Chormodoris quadricolor*, Ga = *G. annulatus*, Ca = *Chormodoris africana*, Jf = *J. funebris*). The only significant differences were between *Chormodoris quadricolor* and *J. funebris* (*p* = 0.01).

**Figure 3:**
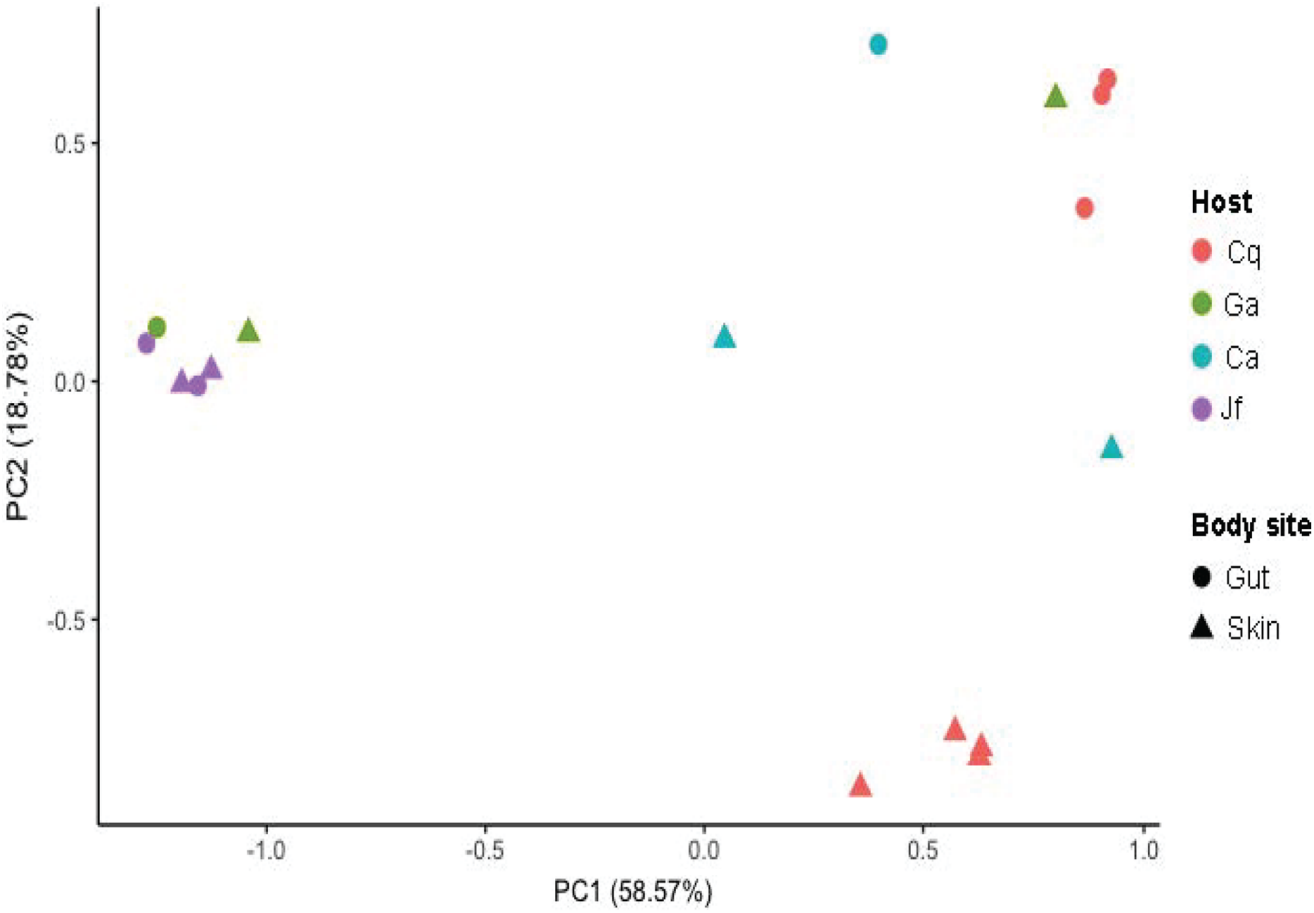
Beta diversity of nudibranch microbiomes. Beta diversity analysis showed samples separated largely by host organism (Cq = *Chormodoris quadricolor*, Ga = *G. annulatus*, Ca = *Chormodoris africana*, Jf = *J. funebris*), with additional separation of gut and skin samples in *Chormodoris quadricolor*.

**Figure 4:**
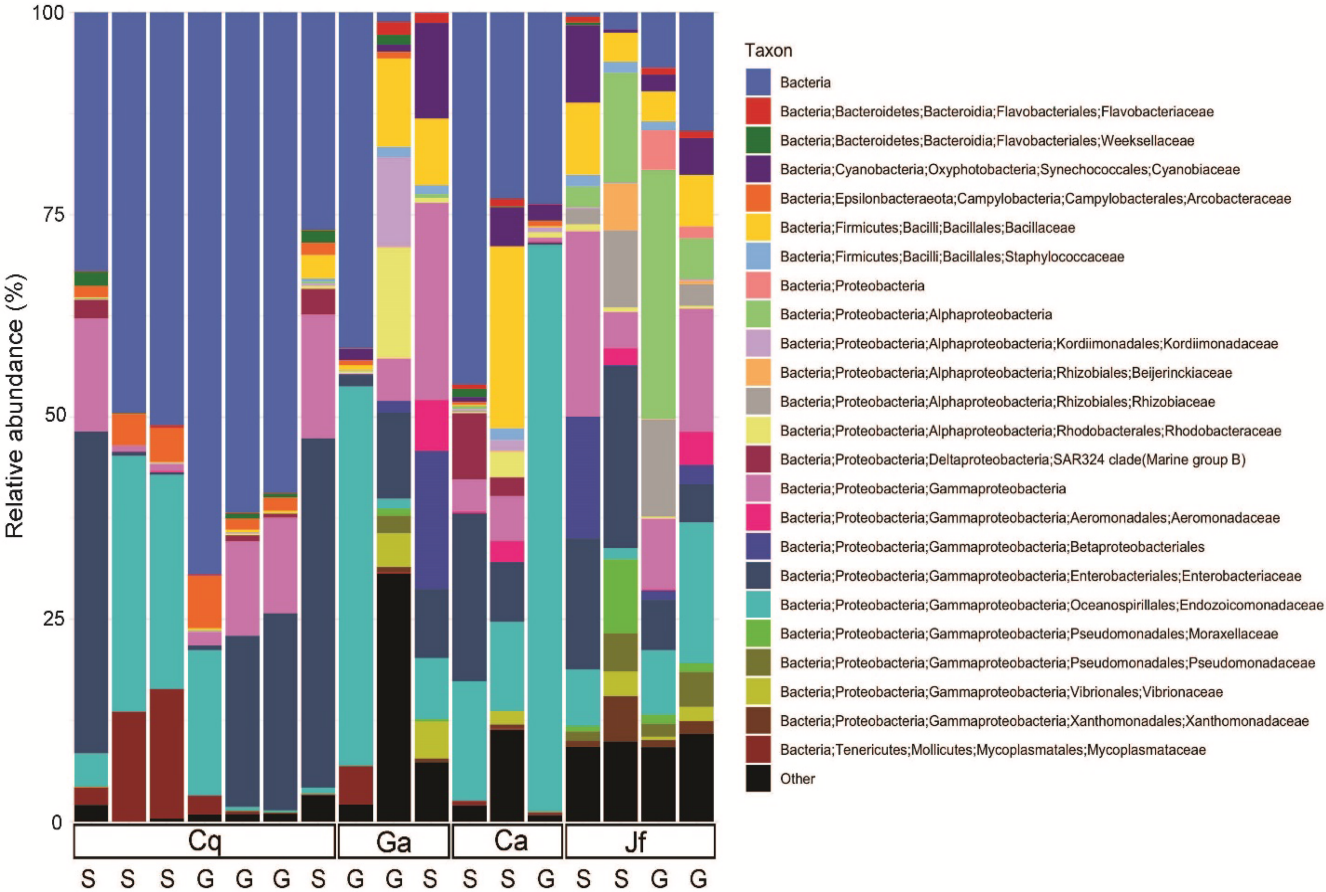
Taxonomic composition of nudibranch microbiomes. Nudibranch (Cq = *Chormodoris quadricolor*, Ga = *G. annulatus*, Ca = *Chormodoris africana*, Jf = *J. funebris*) microbiomes showed a large fraction of SVs, particularly in *Chormodoris quadricolor*, were unclassified beyond the domain level (“Bacteria”). Other well-represented taxa included members of the Gammaproteobacteria and the family Bacillaceae (Firmicutes).

The skin and gut microbiomes of four nudibranchs (*Chormodoris quadricolor*, *G. annulatus*, *Chromodoris africana* and *J. funebris*) were assessed by high-throughput amplicon sequencing of 16S rRNA gene fragments. Alpha diversity analyses suggested that the nudibranch microbiomes were largely captured by the sequencing depth (Fig. S1), which ranged from 1,448 to 21,968 sequences before Deblur processing and removal of chloroplast sequences. While the four specimens had varying levels of diversity, the skin microbiomes were consistently more diverse than the gut microbiomes (Fig. 2). Beta diversity analyses showed separation of samples by both host identity and body part (gut vs. skin), although the patterns were not consistent (Fig. 3). Several microbiomes were dominated by sequences unidentifiable past the domain or phylum level (Fig. 4), particularly in *Chormodoris quadricolor*. One notable finding was the prevalence (between 20% and 60% sequences) of two sequences with no close relatives. These sequences shared ~92% identity unidentified Bacteria and unidentified Gammaproteobacteria, and were found in the microbiome sequences from most of the nudibranch tissue samples. The presence of these sequences in holobiont DNA was confirmed by PCR with specific primers, followed by Sanger sequencing (Table 5). Attempts to obtain longer sequences using primers 331F and 797R were partially successful. The longer 16S rRNA gene PCR products had ~92% identity with three uncultured bacteria, suggesting potentially novel taxa (Table 5). Other well-represented families in the microbiomes included *Endozoicomonadaceae* (Gammaproteobacteria), *Enterobacteriaceae* (Gammaproteobacteria) and *Bacillaceae* (Firmicutes) (Fig. 4). Archaea made up only 0.08% of total sequences, while Gram-positive and Gram-negative bacteria represented 2.7% and 97.3% of total sequences, respectively. The relative abundance of the 8 most abundant families (with the exception of *Bacillaceae* and *Enterobacteriaceae*) varied significantly among nudibranch species. The family *Rhizobiaceae* and an unidentified alphaproteobacterial taxon were represented only in *J. funebris*, while the *Mycoplasmataceae* and *Arcobacteraceae* were highest in *Chormodoris quadricolor* and absent in *J. funebris*. Cyanobacteria were higher in *J. funebris* and *G. annulatus* than in *Chormodoris quadricolor* and absent in *Chormodoris africana*. Fourteen Gram-positive genera were present in microbiome data, while only seven were isolated from homogenized animal tissues. Interestingly, while *Rhodococcus* and *Staphylococcus* represent a high number of SVs (79 and 165, respectively), they were a small proportion of the bioactive cultured isolates, one and ten isolates respectively. Similarly, although *Nocardiopsis* represented a small number of SVs (28), they represented 10% of identified cultured isolates (seven isolates).

**Table 5:** Nearest relatives of unidentified 16S rRNA sequences from nudibranch microbiomes.

Unidentified SV taxonomy	Closest 16SrRNA gene NCBI match to amplicon (~250 bp)	Identity (%)	Closest 16SrRNA gene NCBI match to PCR product (~ 700 bp)	GenBank accession no.	Identity (%)
Bacteria	Uncultured *Mollicutes* bacterium clone M15	95.83	Uncultured *Mollicutes* bacterium clone M15	JX966226	92.5
*Gammaproteobacteria* A	Uncultured bacterium clone FW3B7_P4_B07	97.23	Uncultured bacterium clone 6215-B62	HM173271	92.33
*Gammaproteobacteria* B	Uncultured marine bacterium clone 155S3Bc9Zp	93.69	Uncultured bacterium clone SIP12C_5A	KF741496	92.45

## Discussion

The increasing frequency of antibiotic resistant bacterial infections necessitates the discovery of novel antibiotics to counteract pathogen resistance. Currently, cultivation and fermentation of microorganisms isolated from the marine environment is one of the most important sources for novel pharmaceuticals (Hughes & Fenical, 2010; Blockley et al., 2017). Exploring understudied sources of novel microbes, such as nudibranch microbiomes, may provide an exploitable resource for drug discovery.

The prolific chemical biology of nudibranchs has been well documented (recently reviewed in Dean & Prinsep (2017)). Many studies have focused on the origin of the secondary metabolites: either de novo synthesis by the nudibranch (Kubanek, Graziani & Andersen, 1997; Kubanek & Andersen, 1999; Barsby, Linington & Andersen, 2002; Fontana et al., 2003), or derived from a food source, sometimes followed by transformation by the nudibranch (Gavagnin et al., 2004; Manzo et al., 2007). The dorid nudibranchs used in this study fall into two families, the Chormodorididae (*Ceratosoma trilobatum*, *Chormodoris quadricolor*, *Chormodoris africana* and *G*. *annulatus*) and the Discodorididae (*J*. *funebris*). There are relatively few studies regarding the natural products from these organisms. Australian specimens of *Ceratosoma trilobatum* were shown to possess different furanosesquiterpenes in the mantle compared to the viscera (Winters et al., 2018), and deterrent furanosesquiterpenes were found to defend Indo-Pacific animals (Mollo et al., 2005). Early reports of *Chormodoris quadricolor* from the Red Sea describe the presence of the sponge natural products latrunculin A and B (Ilan, 1995), as *Chromodoris* spp. feed exclusively on sponges, and use the sponge metabolites for defense (Corley et al., 1988). There are no reports of metabolites found in *Chormodoris africana* or *G*. *annulatus*. *Jorunna funebris* has been shown to sequester bioactive isoquinoline quinones from its sponge prey (Huang et al., 2016), although another active metabolite, jorumycin (Fontana et al., 2000), was not found in prey sponges. While it is clear that many nudibranchs have diverse mechanisms for obtaining defensive chemicals, including exploiting their prey metabolites, the prevalence of microbial symbiont-produced metabolites in other marine invertebrates suggests that the nudibranch microbiome may also contribute to their prolific chemistry (Dean & Prinsep, 2017).

Few studies have addressed the natural product biosynthetic potential of microbes associated with nudibranchs (with the exceptions of (Riyanti, Widada & Radjasa, 2009; Bohringer et al., 2017; Kristiana et al., 2020)). As many natural products come from Gram-positive bacteria, this type of microbe was targeted for isolation and characterization. All 16S rRNA-identified Gram-positive isolates cultured from the homogenized animal tissues were present in the microbial community analysis and assigned to Firmicutes (mainly *Bacillus* spp.) and Actinobacteria (particularly the genera *Streptomyces*, *Nocardiopsis* and *Kocuria*) (Table 4). *Streptomyces* spp. and *Nocardiopsis* spp. cultured from the homogenized animal tissues represent 7 and 19% respectively of the total *Streptomyces* and *Nocardiopsis* SVs in the microbiome analysis. *Kocuria* was present in both the cultured and uncultured microbiota associated with *G. annulatus*, but absent from both in *Chormodoris quadricolor*, suggesting that there may be some host-specificity in the associations between nudibranchs and bacteria. All *Kocuria* isolates harbored PKS and NRPS biosynthetic gene clusters, and their production of antimicrobial compounds (Palomo et al., 2013) suggests that they may play a defensive role in nudibranch chemical ecology.

Associated *Bacillus* spp. may represent a particularly fruitful source of bioactive compounds. Cultured *Bacillus* represent 2.5% of the total bacilli SVs in the microbiome analysis. Interestingly, while *Bacillus* represented a small proportion of the community, they were a high proportion of the bioactive isolates (38%) from *Chormodoris quadricolor*. The genus *Bacillus* currently includes more than 200 described species and many strains are known as producers of antimicrobial compounds, including peptides and lipopeptides (Caulier et al., 2019). All isolated bacilli were present in the host microbial community, with 99–100% sequence identity. Interestingly, one of our isolates, 3ISP2, contained both PKS and NRPS genes, and is closely related to *Bacillus siamensis* strain ICMP 20282. Another *B*. *siamensis* strain (JFL15) was recently shown to produce cyclic lipopeptides with antimicrobial activity against multidrug resistant pathogens (Xu et al., 2018), suggesting that 3ISP2 may be an attractive target for further natural product discovery.

Other taxa known for producing bioactive natural products were represented in the isolates and the microbial community. *Streptomyces* are prolific producers of secondary metabolites, including antivirals, insecticides, pesticides, and herbicides (Watve et al., 2001). All *Streptomyces* isolates exhibited antimicrobial activity and contained PKS and NRPS biosynthetic gene clusters. While a few *Streptomyces* have been shown to associate with invertebrates, especially insects (Currie et al., 1999; Kaltenpoth et al., 2012; Ortega et al., 2019), most inhabit soil and sediment environments. Even though *Streptomyces* spp. are not highly represented in either the isolates or the community analysis, their ability to produce natural products with diverse ecological activity including allelopathy (Vurukonda, Giovanardi & Stefani, 2018), anti-predation (Kaltenpoth et al., 2012), and anti-fouling (Xu et al., 2009), suggests that they may contribute to nudibranch chemical ecology. The genus *Nocardiopsis* is also well known for its ecological versatility and production of a variety of bioactive compounds such as antimicrobial agents, anticancer substances, tumor inducers, toxins, and immune modulators, and has been shown to associate with a variety of invertebrate and vertebrate animals from marine and terrestrial environment (Bennur et al., 2015). All of the *Nocardiopsis* isolates in this study exhibit antimicrobial activity and harbor PKS and NRPS biosynthetic gene clusters. Their representation in both the isolates and microbial community, coupled with their ability to produce natural products with diverse ecological activity including antimicrobial (Kumar et al., 2012), neuroactive and cytotoxic effects (Lin et al., 2013), portends their potential role in nudibranch chemical biology.

To better understand the bacterial community associated with nudibranchs, we coupled traditional cultivation with next generation sequencing. Our analyses suggested that the communities associated with the four nudibranchs were different, even though they were collected from the same environment. In addition, not surprisingly, the 16S rRNA sequences indicate that the animals possess a more diverse Gram-positive community compared to the cultured isolates. For instance, only four genera of actinomycetes were isolated while nine genera were identified though microbial community analysis. Further, 297 actinomycete SVs were identified in the community analysis, but only 20 were isolated. The microbial communities in the nudibranchs displayed higher alpha diversity of the skin microbiome relative to the gut microbiome (Fig. S1), possibly reflecting the presence of microbes acquired from other species or the water column compared to a less-exposed gut community. Gut microbiomes may also face selection pressure from a diet rich in bioactive compounds (Rudman & Bergquist, 2007), possibly inhibiting the growth of many common gut microbes. Further, while it is commonly assumed that invertebrates have a “core” gut microbiome, recent evidence from caterpillars suggests this is not always the case (Hammer et al., 2017). Nudibranch guts may feature primarily transient microbes, which would also explain the high inter-individual variability of both gut and skin microbiota (Figs. 3 and 4). In particular, the large differences between the two *Chromodoris* species suggest there is little consistency in microbiome composition at the animal genus level. Moreover, most samples featured high levels of a SV classified only to the domain Bacteria, suggesting important novel diversity within the nudibranch microbiome (Fig. 4). Other prominent taxa include the families *Endozoicomonadaceae* and *Arcobacteraceae*, both of which are often host-associated microbes. In particular, Endozoicomonads are extremely prevalent in marine invertebrate microbiomes, particularly those of coral (Bayer et al., 2013; Pogoreutz et al., 2018). Sequence data suggested most of the dominant members of the nudibranch microbiome were not captured by cultivation. While members of the phylum Firmicutes were reasonably well-represented in three out of four nudibranchs, Actinobacteria comprised a small fraction of all microbiomes (Fig. 4). These results are similar to those of other studies of marine invertebrates showing wide discrepancies between strain isolation and high-throughput sequencing efforts (Correa et al., 2013). Combined with the high fraction of unidentified bacterial SVs in the sequence data, these results reinforce the hypothesis that as-yet uncultured bacteria may produce novel bioactive compounds and identify nudibranchs as a valuable subject for future metagenomic studies targeting biosynthetic gene clusters. Further, deep pyrosequencing of nudibranch community 16S rRNA allowed us to assess the bacterial taxa that were not cultured, which potentially will allow optimization of growth techniques to increase the likelihood of isolation of strains with potentially novel bioactivity.

## Conclusions

Marine bacteria are an important source for the discovery of unique natural products with novel bioactivity. While considerable effort has gone into the study of many marine invertebrates and their bacterial associations, much less attention has been paid to the microbiomes of nudibranchs, prolific sources of natural products. In this study, Gram-positive bacteria associated with nudibranchs were screened for antimicrobial and antitumor activity, as well as the presence natural product biosynthesis genes. We found that a majority of the isolates tested had bioactivity and possessed natural product biosynthetic gene fragments. Many isolates with multiple types of biosynthetic genes were identified as Firmicutes and Actinobacteria, frequent producers of bioactive compounds. It is not known if these isolates produce compounds that contribute to nudibranch defense, but 16S rRNA community amplicon sequencing indicated all bacterial isolates were present in the uncultured host-associated microbiome, suggesting that they may be ecologically relevant. Most nudibranch specimens featured high levels of a SV classified only to the domain Bacteria, suggesting important novel diversity within the nudibranch microbiome. As nudibranch-associated bacteria have the potential to produce bioactive compounds, future metagenomics and metabolomics studies of these understudied systems may reveal novel biosynthetic gene clusters and natural products to help augment the natural product preclinical pipeline.

## Supplemental Information

10.7717/peerj.10525/supp-1 Supplemental Information 1 Sequencing depth of (A) skin and gut samples from four nudibranch specimens, and (B) from each nudibranch sample.

## Additional Information and Declarations

### Field Study Permissions

The following information was supplied relating to field study approvals (i.e., approving body and any reference numbers): Faculty of Pharmacy Research Ethical Committee, Suez Canal University, approved the study (201511R1).

### Data Availability

The following information was supplied regarding data availability: Nudibranch partial COI nucleotiode data are available at NCBI: MT446216, MT446217, MT639354 and MT659206. Isolate 16S rDNA data are available at NCBI Nucleotide: MT393617–MT393684. Microbiome 16S rDNA data are available at the Sequence Read Archive: PRJNA629797.
